# A developmental explanation of the dependence of binaural best delays on characteristic frequency

**DOI:** 10.1186/1471-2202-12-S1-O10

**Published:** 2011-07-18

**Authors:** Bertrand Fontaine, Romain Brette

**Affiliations:** 1 Laboratoire Psychologie de la Perception, CNRS and Université Paris Descartes, 45, rue des Saints Pères, 75006 Paris, France; 2 Equipe Audition, Département d'Etudes Cognitives, Ecole Normale Supérieure, 29, rue d'Ulm, 75005 Paris, France

## 

Both mammals and birds use interaural time difference (ITD) as a cue to determine the horizontal position of a sound source in space. In the barn owl brainstem, neurons of the Nucleus Laminaris (NL) have firing curves which vary with the acoustic stimulus ITD. Each NL neuron is tuned to a certain ITD in that their response is maximal for a certain ITD, i.e., they respond preferably when the stimuli from the two ears have a certain delay, called best delay (BD) of that neuron. Recently, it has been shown that the distribution of BDs along the tonotopical axis depends on the best frequency (BF) of their corresponding neurons. In particular, BDs rarely exceed 1/(2BF), an approximate constraint called the pi-limit. The origin of this BF-dependent distribution of BDs is still a matter of debate.

In this work, we use a modeling approach to test whether the pi-limit could emerge from activity-driven plasticity, in particular Spike-Timing Dependent Plasticity (STDP). Using a standard peripheral model and physiologically plausible spiking neuron models, we first feed binaural acoustic noise with ITDs in the physiological range of the barn owl (smaller than 250 µs) to NL neurons undergoing STDP and analyze the distribution of the resulting BDs. We show that STDP selects the inputs based on their synaptic delays and that the resulting BD distribution mainly falls between plus or minus half the characteristic period of the corresponding neurons. We then use uncorrelated binaural white noises and show that the ITD selectivity and the dependence of binaural best delays on characteristic frequency also emerges after a simulated developmental period. We finally check that our conclusions also hold in more realistic settings, by feeding the model with real forest recordings.

In conclusion, our results suggest that the frequency-dependence of BDs may simply be a by-product of the way ITD tuning develops in binaural neurons. It does not impair the ability of these neurons to represent the azimuth of sound sources, because BDs that differ by an integer number of characteristic periods are mostly redundant. Perhaps more interestingly, it also provides a complete representation of ITDs which is functional in any acoustical environment, even if the head of the animal continues to grow after the critical development period. This suggests that the distribution of BDs does not simply mirror the statistics of binaural sounds during development, but instead provides a robust representation of changing environments.

